# Influence of Starch Cross-Linking on the Performance of Cellulose Aerogels for Oil Spills Sorption

**DOI:** 10.3390/gels11060386

**Published:** 2025-05-24

**Authors:** Rafael Picazo Espinosa, Jochen Uebe, Marija Katarzyte, Tatjana Paulauskiene

**Affiliations:** 1 Marine Research Institute, Klaipeda University, H. Manto 84, 92294 Klaipeda, Lithuania; rafael.picazo.espinosa@ku.lt (R.P.E.);; 2 Engineering Department, Faculty of Marine Technology and Natural Sciences, Klaipeda University, Bijunu Str. 17, 91206 Klaipeda, Lithuania; jochen.uebe@ku.lt

**Keywords:** cellulose aerogel, cross-linking agent, starch, sorption capacity, oil spill, element mapping, apparent viscosity, petroleum hydrocarbons, bioremediation

## Abstract

Oil spills represent a significant environmental threat due to the toxicity of hydrocarbons, particularly in aquatic environments where oil rapidly spreads across the surface. Sustainable sorbents are needed for an efficient and eco-friendly response to oil spills. Cellulose aerogels produced from recycled paper and cardboard exhibit promising properties such as buoyancy, light weight, biocompatibility, and recyclability. Mechanical stability and reusability can be enhanced using cross-linkers such as starch. This study evaluated the impact of starch on cellulose aerogel morphology, sorption capacity for various petroleum products (crude oil, marine diesel, and lubricating oil), and reusability using scanning electron microscopy (SEM) and elemental mapping. Aerogels containing 0.5 and 1 wt% starch showed higher porosity, sorption capacity, and reusability. Starch did not affect hydrophobization or significantly alter nitrogen and carbon levels, indicating limited influence on surface chemistry and adsorption performance.

## 1. Introduction

Oil spills are a recurring occurrence in our time, when material and energy production is largely based on fossil oil. The release of petroleum hydrocarbons into the environment occurs due to incidents during oil exploration and production, loading operations, or transportation. Some of these pollutant compounds could also leak out when ships are refueling.

The resulting slicks not only change the color, acidity, taste, and smell of water, where they spread forming a thin film more quickly and easily than on land, but, most importantly, they have a toxic effect on aquatic organisms and impact marine ecosystems [1,2,3,4,5]. This environmental damage is intensifying, and its relevance to humanity is growing due to its connection with rising climate change and health hazards. Therefore, sustainable and innovative solutions are needed to address these pollution events [4,6].

In surface spills, sorbents are effectively used for coastal clean-up and the removal of small hydrocarbon slicks [7]. Sorbents are among the most common methods used for this purpose [8,9]. Despite their extensive use, conventional sorbents, such as polypropylene, are not the most sustainable solutions from an economic and environmental standpoint due to the non-biodegradable, non-renewable materials used for their production. Low-cost, abundant, non-toxic, biodegradable, and reusable cellulosic materials could be an alternative and have a significant positive impact on sustainability and the circular economy. In addition, sorbents must have high specificity for the target contaminant to be removed, high sorption capacity, and easy regeneration for reuse [5]. Therefore, it is advantageous to remove the oil as quickly as possible after the spill using sustainable sorbents and degrade it in designated sites via bioremediation. Bioremediation of oil has already been proven in numerous studies [10,11,12] and can be improved with nutrient addition [13].

Aerogel is a unique porous material with properties that fulfill the main criteria for oil spill cleaning sorbents, such as low density (0.003 to 0.500 g cm^−3^), high porosity (80–99.8%), large specific surface area (100–1600 m^2^ g^−1^), and sufficient chemical surface activity. Aerogels are designed to remove oil or organic solvents from water and serve as an alternative to traditional sorbents. Research on aerogels began more than 90 years ago, but significant breakthroughs have occurred in recent decades, when aerogels have been used in the construction sector (for special thermal and sound insulation), water or air purification, the automotive sector (electric vehicle batteries manufacturing), and the space sector. Given recent trends, ecological methods for removing water pollutants are increasingly sought, and for this purpose, aerogels made from natural materials—cellulose-based are utilized [14,15].

To improve the mechanical stability, durability, and functionality of aerogels, cross-linking is used. Cross-linking refers to the formation of three-dimensional networks between cellulose fibers and different cross-linking agents, additives such as the synthetic compounds glutaraldehyde, dimethylsulfoxide, ethylenediamine and polyester resins [16,17], or biocompatible molecules such as alginate, chitosan, dextran, guar gum [18], as well as biodegradable and/or recycled polymers including poly(ethylene glycol), poly(lactic acid), cellulose acetate, and starch [16,17,19,20,21]. Starch is an attractive, green, and sustainable cross-linker due to its abundance, non-toxicity, renewability, and biocompatibility. It has a branched structure with varying degrees of crystallinity and contains multiple hydroxyl groups that can interact with cellulose fibers and aid in pollutant adsorption. Additionally, it does not require organic solvents, complex protocols, or purification steps after use as a crosslinker [20,21]. Cross-linking can be achieved through physical or chemical methods. Physical cross-linking depends on reversible interactions among the cross-linker and cellulose, such as hydrogen bonds and electrostatic forces, while chemical cross-linking forms covalent bonds between the -OH groups of cellulose molecules and reactive groups of the cross-linkers [19]. This study investigates the impact of starch cross-linking on the chemical, physical, and mechanical properties of cellulose aerogels designed for cleaning up aquatic oil spills. The cellulose aerogel samples examined in this work consist of randomly distributed fibers with non-reproducible arrangements and weak inter-fiber connections. In this context, starch functions as a cross-linking agent, binding the loosely connected cellulose fibers together. Various starch concentrations were employed to explore how compositional differences influence aerogel properties and to gain a deeper understanding of the cross-linker in the aerogel production process.

## 2. Results and Discussion

### 2.1. Aerogels Physical Properties

#### 2.1.1. Density and Porosity

The cellulose aerogel samples had densities ranging from 0.037 to 0.080 g cm^−3^. The mean density of the samples with 0.5 wt% starch was 0.044 ± 0.006 g cm^−3^, with 1 wt% starch was 0.046 ± 0.002 g cm^−3^, and with 3 wt% starch was 0.067 ± 0.004 g cm^−3^. The porosity of the cellulose aerogel samples was higher than 97%. There was a statistically significant difference in mean porosity percentage between at least two starch levels (*p* = 0.00) (Table S1). For aerogel samples with 0.5 wt% and 1 wt% starch, the porosity value was 98%, while for aerogel samples with 3 wt% starch, the porosity value was 97%.

The changes in porosity could be explained by the higher degree of cross-linking of the cellulose fibers by the starch molecules in 3 wt% starch aerogels and the expansion of ice crystals in the larger-sized pores during the freezing step of aerogel production. This leads to the formation of a smaller number of pores with larger diameters and thicker walls after freeze drying, compared to the more abundant but narrower pores with thin walls observed for the 0.5 wt% and 1 wt% starch aerogels, as will be discussed in Section 2.2.1 and Section 2.2.2. These changes in the diameter of pores and the thickness of the adjacent cellulose walls have been reported in [22] and, as will be discussed in the following sections, have implications for the mechanical resistance of the aerogels, as well as for their sorption capacity and reusability.

#### 2.1.2. Wettability and Maximum Sorption Capacity

**Figure 1:**
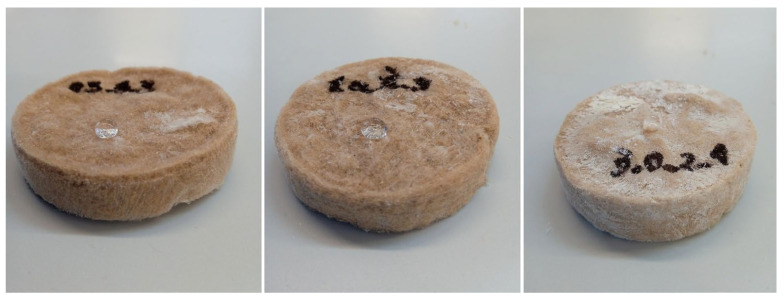
Aerogel samples with 0.5 wt% (**left**), 1 wt% (**center**), and 3 wt% starch (**right**).

All aerogels were hydrophobic, with water contact angles exceeding 90 degrees (Figure 1). The water contact angle varied from 130 to 135 degrees based on the starch concentration in the samples (Figure 1). However, the hydrophobic properties of methyltrimethoxysilane (MTMS) used as a hydrophobizing agent could be limited to the external surfaces of the aerogel samples [23]. The element mapping, which will be discussed in Section 2.2.2, shows that the Si atoms contained in the MTMS appear associated with the edges of fibers and the walls of the pores. This confirms that the hydrophobization of the samples was not limited to the external surfaces, and the MTMS reacted with the cellulose fibers located within the pores throughout the bulk of the aerogel samples.

**Figure 2:**
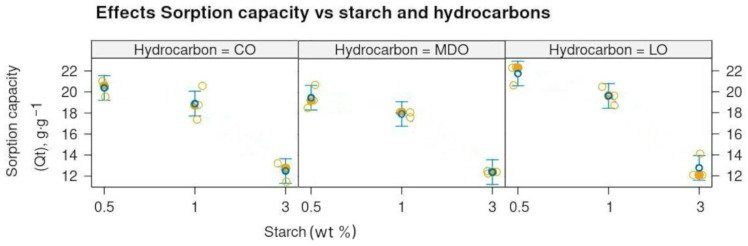
Effects plot illustrating the decrease in sorption capacity as starch percentage increases. Clear circles correspond to the replicates, filled circles correspond to the mean.

No significant differences were observed in the maximum sorption capacity across the different oil products. However, the sorption capacity was strongly (*p* < 0.01) influenced by the starch concentration in the samples. For samples with 0.5 wt% starch, the maximum sorption capacity was 20.545 ± 1.137 g g^−1^ (Table S2). This decreased by approximately 10% for samples with 1 wt% starch, with a mean maximum sorption capacity of 18.824 ± 0.840 g g^−1^. For samples with 3 wt% starch, the sorption capacity was reduced by up to 41%, with a value of 12.782 ± 0.463 g g^−1^, compared to the 0.5 wt% starch samples (Figure 2). Refs. [24,25] reported in their study of milk packages recycled cardboard aerogels that the formation of smaller pores is related to the higher density of the aerogels produced from a higher concentration of cellulose, which reduces the available space for the growth of ice crystals during the freezing step of the aerogel production process. A similar explanation can be applied to samples with higher starch content, as the increased starch content occupies the available free volume, leaving less space for sorption. In the present study, the aerogels with lower concentrations of starch (0.5 and 1 wt%), due to the moderate cross-linking level of the cellulose fibers and the small size of the pores induced by the higher cellulose density, can withstand higher amounts of hydrocarbons into their narrower pores while retaining enough elasticity and structural integrity to recover after squeezing. In contrast, the aerogel samples with a higher percentage of starch (3 wt%) hold lower amounts of hydrocarbons in their wider and less flexible pores [25].

#### 2.1.3. Reusability, Sorption Capacity, and Oil Recovery Rate

Preliminary tests beyond five sorption–squeezing cycles revealed structural damage and disintegration of the aerogels, which is expected given their fully natural, biodegradable composition. Therefore, five cycles represent a practical and meaningful reusability threshold for small-scale oil spill applications.

**Figure 3:**
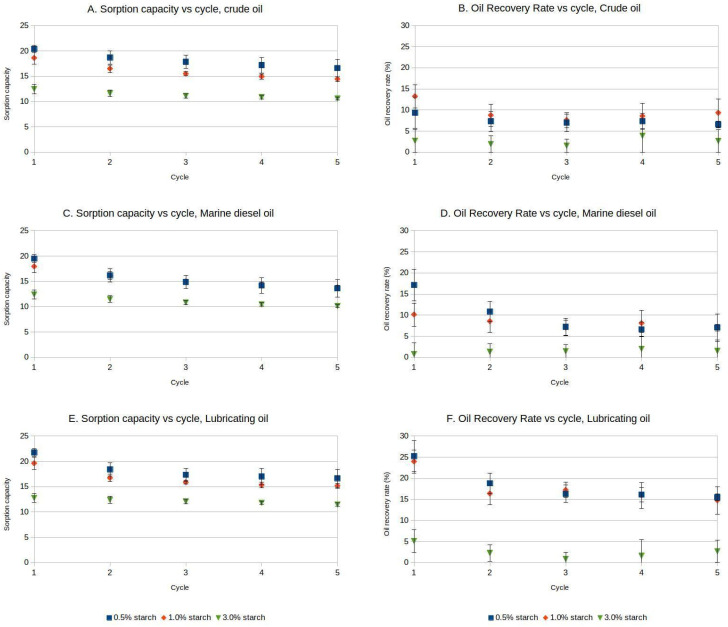
Sorption capacity and oil recovery rate changes with aerogel reuse cycles. (**A**,**B**)—Crude oil; (**C**,**D**)—Marine diesel oil; (**E**,**F**)—Lubricating oil. Mean values and standard deviations are presented for three replicates.

After five cycles, the samples remained visually and mechanically stable and retained their ability to adsorb hydrocarbons for several additional cycles. Refs. [26,27] describe the reasons for the loss of compressibility of aerogel samples as in this paper: progressive buckling of the cell walls and their collapse until, at high compressions, the cell walls finally touch, and the fragments condense into a block. Nonetheless, the sorption capacity decreased after each reuse cycle for the different aerogels, with a reduction ranging from 15% to 22% for crude oil after five cycles, 18% to 30% for marine diesel oil, and 10% to 23% for lubricating oil. The oil recovery rate from aerogels with different starch concentrations varied after each cycle, up to five cycles, averaging between 5% and 13% (Figure 3A,C,E).

Two-way ANOVA analyses were performed for the different oil products, with sorption capacity or oil recovery rate as response variables, and starch percentage and reuse cycle as factors (Tables S3–S8). For crude oil, significant differences were found on the sorption capacity related to the different cycles (F = 16.453; *p* = 0.000) and starch percentages (F = 200.500, *p* = 0.000) (Table S3), but there was no interaction between the two factors. Similarly, significant differences were found in oil recovery means related to sorption cycle (F = 3.003, *p* = 0.034) and for starch percentage (F = 6.767, *p* = 0.004), with no interaction between the two factors. For 0.5 wt% and 1 wt% aerogels, the sorption capacity values were similar and showed a similar decay with additional reuse cycles. For 3 wt% starch aerogels, the sorption capacity was relatively stable, but markedly lower than for the other aerogel formulations. The decay of oil recovery rate showed a similar pattern, although the higher standard deviations for this parameter suggest that the complex mixture of hydrocarbons contained in crude oil does not migrate homogeneously into the aerogel matrix (Figure 3A,B).

For marine diesel oil, significant differences were found for sorption capacity regarding starch percentage (F = 413.762, *p* = 0.000) and reuse cycle (F = 96.576, *p* = 0.000), with a strong interaction between the two factors (F = 6.465, *p* = 0.000). Significant differences were observed for oil recovery rate related to reuse cycle (F = 26.114, *p* = 0.000) and starch percentage (F = 17.986, *p* = 0.000), with a strong interaction between the two factors (F = 10.190, *p* = 0.002). Similar to crude oil, the marine diesel oil sorption values of the 0.5 wt% and 1 wt% starch aerogels were similar and decayed after each reuse cycle, while the 3 wt% aerogels showed a lower but more steady sorption capacity value. The oil recovery rate for marine diesel oil followed trends that were similar to those observed with crude oil, although the higher initial values and lower deviations for 0.5 wt% and 1 wt% starch aerogels suggest a more efficient sorption of marine diesel oil (Figure 3C,D).

For lubricating oil, significant differences were found for sorption capacity related to starch percentage (F = 413.762, *p* = 0.000) and reuse cycle (F = 96.576, *p* = 0.000). A strong interaction between both factors was observed (F = 6.465, *p* = 0.00). For oil recovery rate, there were significant differences related to starch percentage (F = 36.632, *p* = 0.000) and reuse cycle (F = 7.997 *p* = 0.000), but the interaction of both factors was weaker (F = 3.099, *p* = 0.011). Similarly to the aforementioned results, the 0.5 wt% and 1 wt% starch aerogels showed similar decreasing sorption capacity and oil recovery rate values over the number of reuse cycles, while the 3 wt% starch aerogels presented a lower but relatively steady sorption capacity and oil recovery rate (Figure 3E,F). The higher initial values of oil recovery rate of 0.5 wt% and 1 wt% starch aerogels for lubricating oil, compared to the other tested hydrocarbons, as well as the smaller deviations observed in the measurements, suggest that lubricating oil migrates better through the pores of the aerogels during the sorption and desorption processes. These results align with the consulted literature. Ref. [23] indicated that the sorption capacity of hydrophobic cellulose aerogels and filter papers generally increases for oils with lower density.

Despite the observed decay with the reuse cycles, the sorption capacities of the different tested aerogels for marine diesel oil, crude oil, and lubricating oil, even after five reuse cycles, were in range or higher than those reported for similar petroleum products with different hydrophobic cellulose-based sorbents [3,26,28,29]. Likewise, the results, similar to those described in [30], appear to be the result of a combined effect of the cellulose fibers and the starch network [31]. Starch dissolved in water forms a hydrogel, which, after freeze-drying, forms an aerogel. In this case, the starch aerogel could be described as a biocompost filled with cellulose fibers with an aerogel structure. The same applies vice versa. In each case, the properties of the respective pure aerogel appear to be transferred to the aerogels described here, thus enhancing the sorption capacity for hydrocarbons. At approximately 3 wt% cellulose content, the advantage appears to diminish.

### 2.2. Morphological and Structural Characterization

#### 2.2.1. Morphological Characterization

**Figure 4:**
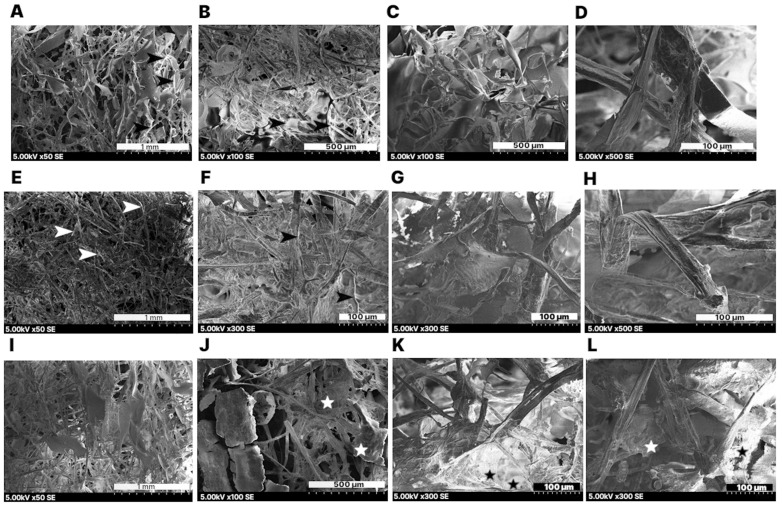
Scanning electron microscopy (SEM) images of cellulose aerogel samples with 0.5 wt% starch (**A**–**D**), 1 wt % starch (**E**–**H**), and 3 wt% starch (**I**–**L**). Bright structures attributed to starch indicated by arrowheads. Precipitated starch zones indicated by stars.

The SEM images showed, in addition to the coarser cellulose fibers, finer, very bright structures that can be attributed to starch (Figure 4A,B,E,F, arrowheads). The exposed groups of these fine structures can interact with the cellulose fibers, contributing to the cross-linking and improv mechanical properties in aerogels. In this regard, ref. [20] reported the interaction of starch with cellulose fibers by strong H-bonds, which they confirmed by Fourier transform infrared spectroscopy (FTIR) analyses.

The starch was molecularly dissolved when added to the cellulose slurry before freezing. This was not self-evident from the scientific literature [32,33]. There, starch is described as grains in dry form that, depending on their composition, either only swell in hot water or are also partially soluble. The composition depends on the proportions of amylopectin and amylose, two forms of starch. Amylose is a form of starch in which the repeating units α-D-glucose are present in a linear molecule. Amylose pectin also has branching of the chain. The branches make starch molecularly soluble in hot water and allow rapid dissolution, while amylose requires higher temperatures and more time to dissolve and can also precipitate when cooled. The latter appears to have occurred in the formulation with 3 wt% starch. Non-fibrous, granular areas characteristic of powdered materials were visible (Figure 4J–L, stars). Ref. [34] reported a more fibrous structure for cellulose-starch aerogels with lower concentrations of starch, and lamellar structures for samples with higher starch concentrations.

#### 2.2.2. Structural Characterization

**Figure 5:**
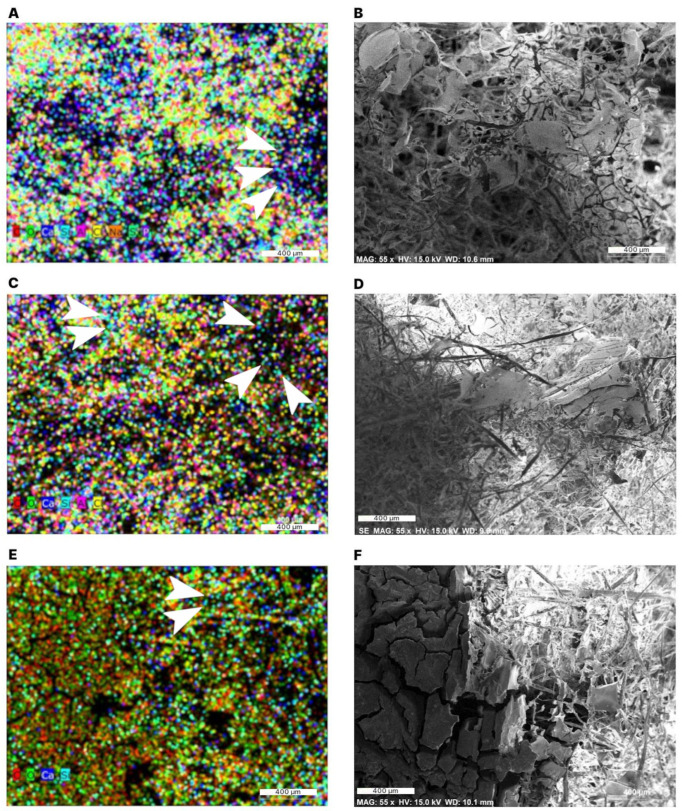
Element maps for aerogels with 0.5 wt% starch (**A**,**B**), 1 wt% starch (**C**,**D**), and 3 wt% starch (**E**,**F**) obtained by EDS. Carbon (C, red), oxygen (O, green), calcium (Ca, dark blue), silicon (Si, light blue, arrow heads), aluminum (Al, pink), chlorine (Cl, yellow), phosphorous (P, purple) and sodium (Na, orange).

During the observation of the sample morphology using scanning electron microscopy (SEM), element mapping can be performed using X-ray diffraction techniques (energy dispersive spectroscopy, EDS) to analyze the chemical composition in regions of interest [35,36,37,38,39,40,41]. The element mapping of the samples showed signals from carbon (C, red), oxygen (O, green), calcium (Ca, dark blue), silicon (Si, light blue), aluminum (Al, pink), chlorine (Cl, yellow), phosphorous (P, purple) and sodium (Na, orange) (Figure 5). The aluminum signal originates from the aluminum-made sample holder. The presence of sodium, phosphorus, chlorine, and calcium can be explained by the tap water used for the preparation of the aerogel samples.

The distribution of colored points in the elements maps and the corresponding SEM image shows that the elements are associated with both the fibers and the “films” between the fibers. However, since the depth resolution is limited to 2 μm, an exact assignment of the colored points to a particular structure in the SEM image could not be made. Nonetheless, the black areas of the element maps can be correlated with the black areas in the SEM images, since the structures were outside the focal plane of the electron beam in both images.

The high signals of carbon and oxygen were expected, since cellulose, the main compound of the samples, is composed of carbon, hydrogen, and oxygen. The method used cannot detect Hydrogen. The presence of Si relates to the methyl silyl group (CH_3_Si), which was formed by the hydrophobization of the aerogel samples with MTMS. The signal intensity of Si was lower than that of carbon and oxygen in the 0.5 wt% starch aerogels (Figure 5A,B), probably because methyl silyl groups coat fiber surfaces during the hydrophobization process. Despite the lower Si signal, the higher hydrocarbons sorption capacity and reusability observed with 0.5 and 1 wt% starch aerogels compared to the 3 wt% aerogels indicate that the functionality of aerogels is determined not only by the degree of hydrophobization and mechanical reinforcement by starch, but also by the geometry of their different internal structures [20,25,35,36].

### 2.3. Analyses of Nitrogen and Carbon Contents of Aerogels

**Figure 6:**
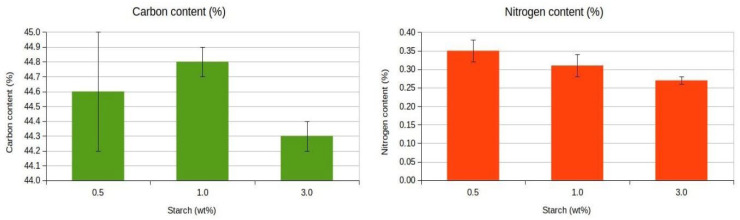
Nitrogen and carbon contents of cellulose aerogels. Mean ± standard deviation based on triplicate measurements.

Nutrients can be limiting factors in the use of sorbents for oil spill bioremediation processes, due to the limited availability of nitrogen in petroleum products, and the low biodegradability of the long and branched chain hydrocarbons and aromatic compounds, which makes them ineffective as carbon sources. Previous studies by our group and others have shown that natural and modified sorbents can biostimulate native microbiota either by releasing nutrients inherent to their composition or by providing a biocompatible solid substrate. This stimulation may support microbial communities capable of degrading hydrocarbons or may influence microbial dynamics by promoting competition for oil hydrocarbons and limited nutrients. Therefore, it is beneficial when the cross-linkers added to the sorbents act as nitrogen and carbon sources and contribute to the biostimulation of oil-degrading microorganisms [11]. The nitrogen content in aerogels with different starch concentrations ranged from 0.35 ± 0.02% for the 0.5 wt % aerogels to 0.27 ± 0.01% for the 3 wt% aerogels. The carbon content ranged from 44.6 ± 0.4% for 0.5 wt% aerogels to 44.3 ± 0.1% for 3 wt% aerogels (Figure 6).

A two-way ANOVA analysis was performed for nitrogen content with starch percentage and carbon percentage as factors (Table S9). A statistically significant difference (F = 12.915, *p* = 0.016) in mean nitrogen content was observed for starch percentage, but no interaction between the starch percentage and the carbon content factors was detected. Additionally, a two-way ANOVA was performed for carbon content, with starch percentage and nitrogen content as factors (Table S10). There were no significant differences for the carbon content regarding starch percentage (F = 4.383, *p* = 0.090), and no interaction of nitrogen and starch percentage was observed. Since cellulose constitutes the majority of the aerogels, and carbon is the main element in cellulose, the addition of small amounts of starch has minimal effect on the aerogels’ carbon content. However, the presence of residual amino acids from the proteins associated with the starch natural sources could, to some extent, increase the nitrogen content of the aerogels. Nonetheless, the lower nitrogen content of the aerogel with 3 wt% starch suggests that the nitrogen contribution of starch added to cellulose aerogels was minimal.

### 2.4. Compressive Mechanical Properties

The Young’s modulus of elasticity reported by [28] for oxidized and silanized aerogels is in range with our results (Table 1), suggesting that the cross-linking of the mechanical strength of the aerogel structure via comparable physical and chemical mechanisms to those observed in more complex treatment methods. Nevertheless, the positive effects of cross-linker addition appear to reach a threshold. Ref. [22] reported an improvement in the mechanical strength of regenerated cellulose fibers with the addition of various co-solvents of up to 10 wt%, however, a decline in performance was noted when the co-solvent concentration exceeded this threshold.

**Figure 7:**
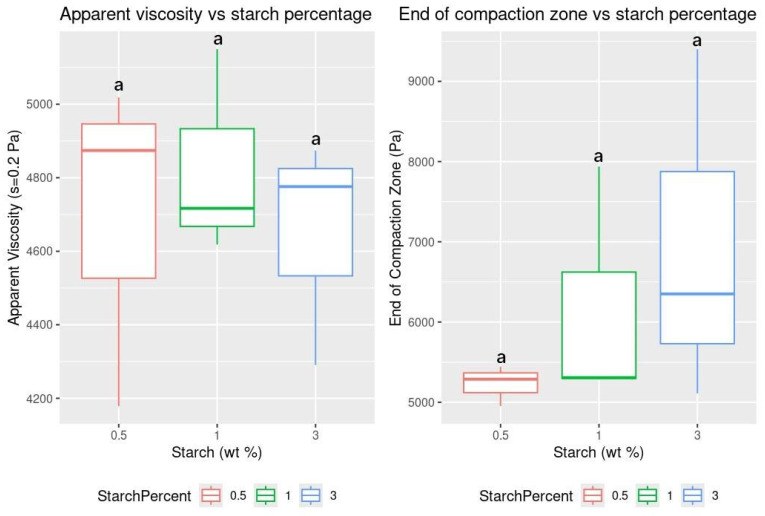
Boxplots for apparent viscosity and end of compaction zone vs. starch percentage. Letters over the boxes indicate grouping based on significant differences.

There were no statistically significant differences in apparent viscosity (Table S11) or in the end of the compaction zone (Table S12) across the different starch concentrations tested (Figure 7). Kim et al. (2024) [38] reported that incorporating co-solvents during cellulose fiber production reduced the viscosity. The co-solvents likely interfered with the hydrogen bonding between the cellulose chains, disrupting their connections. Although no statistically significant differences in apparent viscosity and compaction zone endpoint were observed, the crystallinity of cellulose fibers due to starch disrupting their parallel alignment, as noted in [20], may account for the reduced sorption capacity and reusability of 3 wt% starch aerogels compared to 0.5 and 1 wt% samples. The morphology observed in the SEM images of both studies correlates with our maximal sorption capacity and reusability results and is consistent with findings from similar studies [20,37,38,40]. Therefore, it can be inferred that further increasing the starch content would not improve the overall performance of the cellulose aerogels for their intended use.

## 3. Conclusions

The porosity of hydrophobic cellulose aerogels was affected by the starch percentage, showing greater porosity in 0.5 and 1 wt% starch samples relative to 3 wt% ones.

All aerogel samples underwent consistent hydrophobization, as silicon atoms originating from MTMS were found on the surfaces of the pore walls. The 0.5 and 1 wt% starch aerogels revealed a cross-linked structure, characterized by pores with walls of cellulose fibers and starch network. Aerogels with 3 wt% cellulose showed a higher density of cellulose fibers within the walls. Due to their narrow pores, the aerogels with 0.5 and 1 wt% starch successfully retained more hydrocarbons than 3 wt% starch aerogels, effectively adsorbing and releasing the hydrocarbons while preserving functionality after 5 cycles of use and squeezing.

The addition of starch had little impact on the nitrogen and carbon content in the hydrophobic aerogels.

It can be concluded that the presence of starch in the cellulose aerogel has an effect on the porosity and, therefore, on the sorption of oil at cellulose contents below 3 wt%. Above 3 wt% cellulose content in the aerogel sample, the effect of starch decreases. The intended effect of improving the mechanical stability of starch on the loose network of cellulose fibers cannot be clearly stated based on the observed results.

One recommendation to improve mechanical strength could be to investigate and compare the use of other materials, such as those described in the introduction. Gelatin could be a promising material because it would dissolve in water and provide the nutrients necessary for bioremediation, such as organic nitrogen.

In addition, an analysis of the pore size distribution by analyzing the sorption time could provide further insights into the functioning of the cellulose aerogel, especially if this analysis were performed in a material-dependent manner with respect to the crosslinker.

## 4. Materials and Methods

### 4.1. Chemicals and Materials

Cardboard waste (4.01.00 code according to EN 643:214) and potato starch (Sigma-Aldrich Chemie GmbH, Taufkirchen, Germany) were used as raw materials for the preparation of the aerogels. Methyltrimethoxysilane (MTMS) (98%; Sigma-Aldrich Chemie GmbH, Taufkirchen, Germany) was used for hydrophobizing the aerogels.

To evaluate the sorption efficiency of the aerogels, three petroleum-based products were tested: Arabian Light Crude Oil (CO) with a density of 855 kg m^−3^ and dynamic viscosity of 0.0098 Pa s from SC ORLEN Lietuva (Mazeikiai, Lithuania), Marine Diesel Oil (MDO) with a density of 852 kg m^−3^ and dynamic viscosity of 0.0024 Pa s from JSC Gindana (Klaipeda, Lithuania), and the Lubricating Oil PEMCO iDrive 105 SAE 15w-40 with a density of 877 kg m^−3^ and dynamic viscosity of 0.0038 Pa s from JSC SCT Lubricants (Klaipeda, Lithuania).

### 4.2. Synthesis Procedure for Aerogels

The synthesis of aerogels was conducted following the methodology described in [25], with minor modifications. Briefly, cardboard was shredded into pieces with sides no longer than 1 cm^2^. Six grams of shredded cardboard were homogenized in 154 mL of distilled water in a 600 mL beaker at 20,000 rpm using an UltraTurrax T25 digital dispenser (IKA) equipped with an 18 mm stainless-steel rotor/stator, producing a homogeneous slurry. To ensure complete immersion of the rotor and to obtain the intended final composition, cellulose and starch were initially dispersed at twice their final concentrations in twice the amount of hot water. The final aerogel composition was formulated with 3 wt% cellulose and starch at 0.5, 1, and 3 wt%. The homogenized slurry was poured into 40 mL metal cups and kept at minus 18 °C for 3 hours to induce freezing. Frozen samples were freeze-dried under vacuum at 0.015 hPa with a condenser temperature of minus 105 °C using a ScanVac CoolSafe (LaboGene, Lillerod, Denmark).

### 4.3. Analysis of Aerogels’ Physical Properties

#### 4.3.1. Determination of Density and Porosity

**Figure 8:**
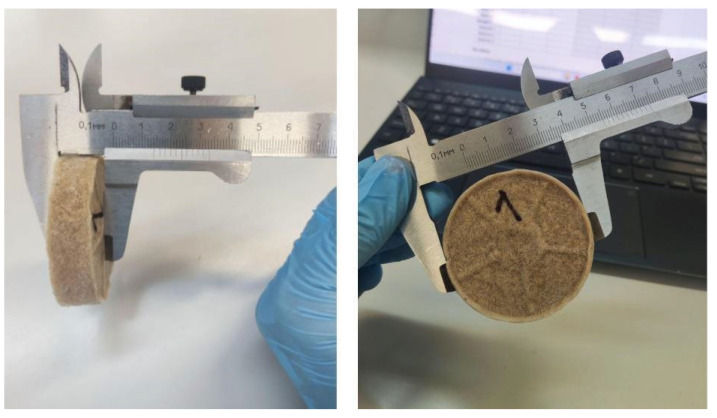
Photographs of aerogel height and diameter measurements.

The weight of the aerogel samples was determined using an analytical balance, and their dimensions were measured with a caliper (Figure 8). The volume of each sample was determined using the SolidWorks software (version 2024). Each measurement was repeated three times. The sample diameters ranged from 5.3 to 5.7 cm, and heights ranged from 0.9 to 1.6 cm.

The relative density of the cellulose aerogel (*ρ_aerogel_*) was calculated as the ratio of the sample weight to the sample volume.

Porosity (*P*) was determined based on the skeletal density of cellulose (*ρ_cellulose_* = 1.5 g cm^−3^; [25,26] and starch (*ρ_starch_* = 0.8 g cm^−3^), using the following formula [26]:

(1) $$P=\left(1-\frac{\rho_{aerogel}}{\rho_{Cellulose}+\rho_{starch}}\right)\cdot 100\%,$$

#### 4.3.2. Wettability Measurement

The wettability of the aerogel surface was assessed by measuring the water contact angle using the sessile drop method. During this process, a small drop of water was placed on the aerogel surface, and a high-resolution camera acquired a close-up image. The contact angle was determined using the ImageMeter tool (ImageMeter GmbH, Braunschweig, Germany), which measured the angle between the solid surface and the tangent of the liquid drop at the solid–liquid interface [26,41,42].

#### 4.3.3. Maximum Sorption Capacity Measurement

Before the experiment, the aerogel samples were weighed. Each sample was then immersed in 250 mL of oil (Crude Oil, Marine Diesel Oil, or Lubricating Oil) for 1 min to ensure adsorption equilibrium was reached [24,41]. After immersion, the samples drained for 1 min and were weighed again. The maximum sorption capacity (*Qt*) was calculated using Formula (2) [41]:

(2) $$Qt=\frac{mw-md}{md}$$

where *Qt* is the sorption capacity at time *t* (in g g^−1^), *md* is the dry weight of the aerogel before sorption (in grams); *mw* is the wet weight after sorption (in grams). The maximum sorption capacity (*Qt*) of the aerogels was measured for three types of oils: crude oil, marine diesel oil, and lubricating oil. Each experiment was performed in triplicate for each hydrocarbon type.

#### 4.3.4. Reusability Test and Oil Recovery Rate Analyses

The reusability of the aerogels was assessed by repeated manual squeezing cycles. After each sorption, the aerogels were manually squeezed to remove absorbed oil, weighed, then re-immersed in the oil for 1 min. This process was repeated for up to five cycles. Three replicates were performed for each oil type.

#### 4.3.5. Morphological and Structural Characterization

A scanning electron microscope (Hitachi S-3400N, Tokyo, Japan) was used to analyze the microstructure of aerogels containing 0.5, 1, and 3 wt% starch. The SEM was equipped with energy-dispersive X-ray spectroscopy (EDS), which included a Bruker Quad 5040 EDS detector, thereby enabling elemental mapping to identify the spatial distribution of starch and other constituents within the cellulose aerogel matrix.

#### 4.3.6. Analysis of Nitrogen and Carbon Content of Aerogels

Before analyzing the nitrogen and carbon content, the cellulose aerogel samples were ground into small fractions using a laboratory mill (IKA A-10 basic mill) and analyzed at the Lithuanian Research Centre for Agriculture and Forestry. Total nitrogen and carbon contents were determined by dry combustion using a CHNS-O elemental analyzer (Costech Instruments Elemental Combustion System 4010, Milan, Italy). Each measurement used 5 mg of ground aerogel, which was placed into a tin capsule and introduced into the analyzer’s autosampler. The gas flow rates were set to 110 mL min^−1^ each for helium and nitrogen, and 180 mL min^−1^ for oxygen. Furnace temperatures were 960 °C and 700 °C, with an oven temperature of 70 °C. Data were processed using Elemental analysis software.

#### 4.3.7. Compressive Mechanical Properties (Mechanical Tests)

The compressive mechanical properties of the aerogels were analyzed using a Zwick/Roell Z020 testing machine, according to the procedure described in [41]. The height and diameter of the 0.5, 1, and 3 wt% starch samples were measured. The machine’s lower grip remained fixed, while the upper grip was connected to a ball joint to ensure parallel compression. The maximum compression force was determined by compressing the cellulose aerogel samples up to 90%. However, as the aerogels did not fully recover their original height, the maximum compression was adjusted to 70% deformation.

Young’s modulus was calculated using the cross-section method, evaluating the elastic portion of the compression curve at 5% and 10% relative deformations. The apparent yield point was determined when the curve deviated by 0.2% from linear deformation (Figures S1–S3).

#### 4.3.8. Statistical Analysis

All measurements were performed in triplicate, and the results are expressed as the mean ± standard deviation. Statistical analyses were conducted using R version 4.4.2 (31 October 2024) under RStudio 2 April 2024. The normality of the data sets was assessed using the Shapiro test. Linear models were established for the various factors and response variables, and one-way or two-way ANOVA analyses were performed. Effects plots and box-plots were generated using R-base (version 4.4.2) and ggplot2 (version 3.5.1), and scatter-plots were created with LibreOffice Calc v. 6.4.7.2. When significant differences were detected (at significant level of α = 0.95), Tukey’s test and Fischer’s LSD test with Bonferroni adjustment were applied to determine groupings among the treatments.

## Figures and Tables

**Table 1:** Comparison of compressive mechanical properties of different cellulose and starch-based materials.

Cellulose Origin	Production Method	Apparent Viscosity ^1^ (kPa·s)	Reference
Recycled cardboard	Freeze drying (aerogel cross-linked with starch)	4.65–4.83	This study
Bacterial cellulose	Freeze drying (aerogel)	-	[28]
Cellulose pulp	Dissolution and regeneration (fibers)	38 to 174	[38]
Microcrystalline cellulose	Solvent exchange and supercritical CO_2_ drying (aerogel cross-linked with starch)	-	[20]
Potato starch and microcrystalline cellulose	Cross-linking with MBA, gelatinization and freeze drying. Starch–cellulose composite aerogel.	-	[39]
Bacterial cellulose	Solvent exchange, impregnation in MTES and freeze drying (Bacterial cellulose—Si aerogel).	-	[26]

^1^ Apparent viscosity is not frequently reported; however, it was included in this study to take into account the dynamic changes in the cellulose aerogels’ structure.

## Data Availability

All data generated or analyzed during this study are included in this published article.

## Supplementary Materials

The following supporting information can be downloaded at: https://www.mdpi.com/article/10.3390/gels11060386/s1, Table S1. ANOVA table for porosity (%) vs. starch percentage and height., Table S2. ANOVA table for maximum sorption capacity vs. starch % and hydrocarbon, Table S3. ANOVA table for sorption capacity vs. sorption cycle and starch percentage. Crude oil, Table S4. ANOVA table for oil recovery rate vs. cycle and starch percentage. Crude oil, Table S5. ANOVA table for sorption capacity vs. sorption cycle and starch percentage. Marine diesel oil, Table S6. ANOVA table for oil recovery rate vs. cycle and starch percentage. Marine diesel oil, Table S7. ANOVA table for sorption capacity vs. sorption cycle and starch percentage. Lubricating oil, Table S8. ANOVA table for oil recovery rate vs. sorption cycle and starch percentage. Lubricating oil, Table S9. ANOVA table for nitrogen vs. carbon and starch percentages, Table S10. ANOVA table for carbon vs. nitrogen and starch percentages, Table S11. One-way ANOVA for apparent viscosity vs. starch percentage, Table S12: Two ways ANOVA for end of compaction zone vs starch percentage. Figure S1. Compression test curves of aerogel containing 0.5 wt% starch, Figure S2. Compression test curves of aerogel containing 1 wt% starch, Figure S3. Compression test curves of aerogel containing 3 wt% starch.

## Abbreviations

The following abbreviations are used in this manuscript:

MTMS	Methyltrimethoxysilane
CO	Crude oil
MDO	Marine diesel oil
LO	Lubricating oil
