# Supplementary material for: Influence of Starch Cross-Linking on the Performance of Cellulose Aerogels for Oil Spills Sorption
Source: Gels. 2025 May 24;11(6):386. doi: 10.3390/gels11060386 (PMC12191776; doi:10.3390/gels11060386)
Supplement: Supplementary file 1 [file gels-11-00386-s001.zip › gels-3641564-supplementary.pdf]

Supplementary information

Table S1. ANOVA table for porosity (%) vs starch percentage and height

Response: Porosity Perc

	Df	Sum Sq	Mean Sq	F value	Pr(>F)	
Starch Percent	2	7.4074	3.7037	194.4444	2.82e-14	***
Height(cm)	1	0.0896	0.0896	4.7056	0.0416907	*
StarchPercent:Height (cm)	2	0.3993	0.1996	10.4806	0.0006973	***
Residuals	21	0.4000	0.0190			

Signif. Codes: 0 '***' 0.001 '**' 0.01 '*' 0.05 '.' 0.1 ' ' 1

Table S2. ANOVA table for maximum sorption capacity vs starch % and hydrocarbon

Response: SorptCapacityQtgg

	Df	Sum Sq	Mean Sq	F value	Pr(>F)	
StarchPercent	2	317.81	158.905	170.1640	2.037e-12	***
Hydrocarbon	2	9.26	4.628	4.9559	0.01929	*
StarchPercent:Hydrocarbon	4	2.96	0.739	0.7918	0.54569	
Residuals	18	16.81	0.934			

Signif. codes: 0 '***' 0.001 '**' 0.01 '*' 0.05 '.' 0.1 ' ' 1

Table S3. ANOVA table for sorption capacity vs sorption cycle and starch percentage. Crude oil.

Response: SorptCapacityQtgg

	Df	Sum Sq	Mean Sq	F value	Pr(>F)	
SorptionCycle	4	59.77	14.942	16.4530	3.081e-07	***
StarchPercent	2	364.17	182.087	200.4997	< 2.2e-16	***
SorptionCycle:StarchPercent	8	5.56	0.695	0.7658	0.635	
Residuals	30	27.24	0.908			

Signif. codes: 0 '***' 0.001 '**' 0.01 '*' 0.05 '.' 0.1 ' ' 1

Table S4. ANOVA table for oil recovery rate vs cycle and starch percentage. Crude oil.

Response: OilRecoveryRateQsPercent

	Df	Sum Sq	Mean Sq	F value	Pr(>F)	
SorptionCycle	4	81.006	20.252	3.0030	0.033850	*
StarchPercent	2	91.274	45.637	6.7674	0.003752	**
SorptionCycle:StarchPercent	8	13.431	1.679	0.2490	0.977271	
Residuals	30	202.311	6.744			

Signif. codes: 0 '***' 0.001 '**' 0.01 '*' 0.05 '.' 0.1 ' ' 1

Table S5. ANOVA table for sorption capacity vs sorption cycle and starch percentage. Marine diesel oil.

Response: SorptCapacityQtgg

	Df	Sum Sq	Mean Sq	F value	Pr(>F)	
SorptionCycle	4	94.993	23.748	96.576	< 2.2e-16	***
StarchPercent	2	203.490	101.745	413.762	< 2.2e-16	***
SorptionCycle:StarchPercent	8	12.718	1.590	6.465	0.00006845	***
Residuals	30	7.377	0.246			

Signif. codes: 0 '***' 0.001 '**' 0.01 '*' 0.05 '.' 0.1 ' ' 1

Table S6. ANOVA table for oil recovery rate vs cycle and starch percentage. Marine diesel oil.

Response: OilrecoveryRateQsPercent

	Df	Sum Sq	Mean Sq	F value	Pr(>F)	
SorptionCycle	1	145.92	145.924	26.114	0.000007863	***
StarchPercent	1	100.50	100.504	17.986	0.0001236	***
SorptionCycle:StarchPercent	1	56.94	56.940	10.190	0.0027110	**
Residuals	41	229.11	5.588			

Signif. codes: 0 '***' 0.001 '**' 0.01 '*' 0.05 '.' 0.1 ' ' 1

Table S7. ANOVA table for sorption capacity vs sorption cycle and starch percentage. Lubricating oil.

Response: SorptCapacityQtgg

	Df	Sum Sq	Mean Sq	F value	Pr(>F)	
SorptionCycle	4	94.993	23.748	96.576	< 2.2e-16	***
StarchPercent	2	203.490	101.745	413.762	< 2.2e-16	***
SorptionCycle:StarchPercent	8	12.718	1.590	6.465	0.00006845	***
Residuals	30	7.377	0.246			

Signif. codes: 0 '***' 0.001 '**' 0.01 '*' 0.05 '.' 0.1 ' ' 1

Table S8. ANOVA table for oil recovery rate vs sorption cycle and starch percentage. Lubricating oil.

Response: OilrecoveryRateQsPerc

	Df	Sum Sq	Mean Sq	F value	Pr(>F)	
SorptionCycle	4	203.54	50.886	7.9968	0.0001636	***
StarchPercent	2	466.20	233.102	36.6323	0.000000008862	***
SorptionCycle:StarchPercent	8	157.74	19.718	3.0987	0.0113654	*
Residuals	30	190.90	6.363			

Signif. Codes: 0 '***' 0.001 '**' 0.01 '*' 0.05 '.' 0.1 ' ' 1

Table S9. ANOVA table for nitrogen vs carbon and starch percentages.

Response: NitrogenPercentRep

	Df	Sum Sq	Mean Sq	F value	Pr(>F)	
StarchPercentRepWt	1	0.0085714	0.0085714	12.9150	0.01565	*
CarbonPercentRep	1	0.0009049	0.0009049	1.3635	0.29558	
StarchPercentRepWt:CarbonPercentRep	1	0.0006052	0.0006052	0.9119	0.38346	
Residuals	5	0.0033184	0.0006637			

Signif. codes: 0 '***' 0.001 '**' 0.01 '*' 0.05 '.' 0.1 ' ' 1

Table S10. ANOVA table for carbon vs nitrogen and starch percentages

Response: CarbonPercentRep

	Df	Sum Sq	Mean Sq	F value	Pr(>F)	
StarchPercentRepWt	1	0.259286	0.259286	4.3834	0.09048	.
NitrogenPercentRep	1	0.090093	0.090093	1.5231	0.27200	
StarchPercentRepWt:NitrogenPercentRep	1	0.094859	0.094859	1.6036	0.26118	
Residuals	5	0.295762	0.059152			

Signif. Codes: 0 ‘***’ 0.001 ‘**’ 0.01 ‘*’ 0.05 ‘.’ 0.1 ‘ ’ 1

Table S11. One way ANOVA for apparent viscosity vs starch percentage

Response: ApparentViscosity_s0.2Pa

	Df	Sum Sq	Mean Sq	F value	Pr(>F)
StarchPercent	2	53841	26921	0.213	0.814
Residuals	6	758388	126398		

Signif. Codes: 0 ‘***’ 0.001 ‘**’ 0.01 ‘*’ 0.05 ‘.’ 0.1 ‘ ’ 1

Table S12. Two ways ANOVA for end of compaction zone vs starch percentage

Response: EndOfCompactionZonePasdensPa

	Df	Sum Sq	Mean Sq	F value	Pr(>F)
StarchPercent	2	4480138	2240069	0.9253	0.4464
Residuals	6	14524741	2420790		

Signif. Codes: 0 ‘***’ 0.001 ‘**’ 0.01 ‘*’ 0.05 ‘.’ 0.1 ‘ ’ 1

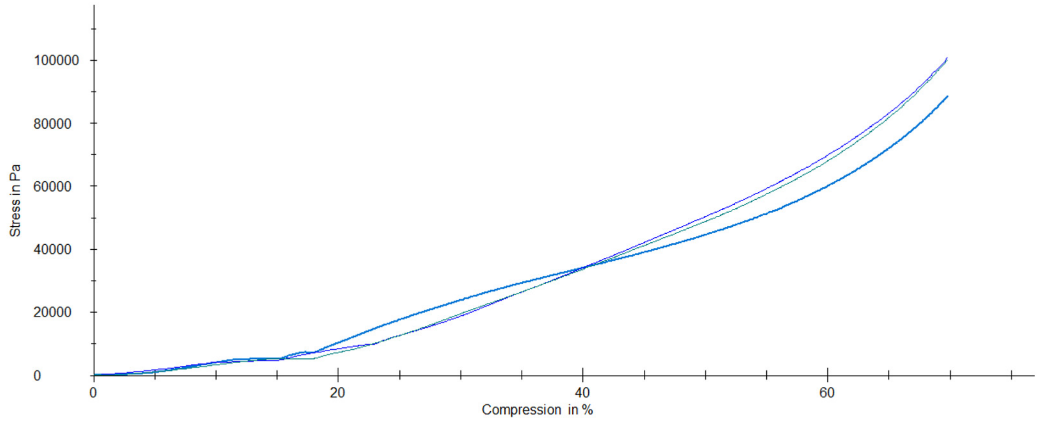

Figure S1. Compression test curves of aerogel containing 0.5 wt% starch. Each curve corresponds to a separate sample analyzed.

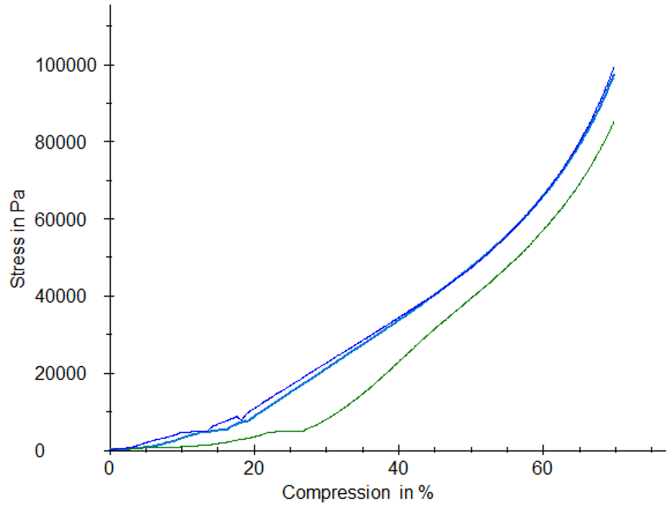

Figure S2. Compression test curves of aerogel containing 1 wt% starch. Each curve corresponds to a separate sample analyzed.

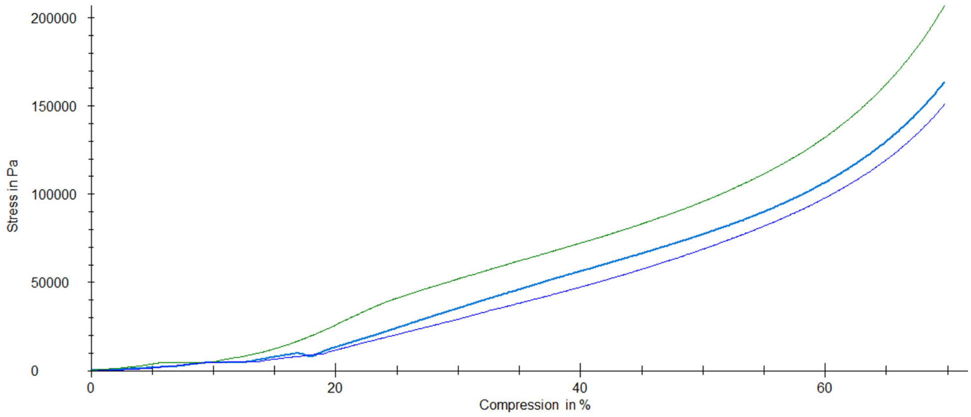

Figure S3. Compression test curves of aerogel containing 3 wt% starch. Each curve corresponds to a separate sample analyzed.
